# Consensus-Based Information Filtering in Distributed LiDAR Sensor Network for Tracking Mobile Robots

**DOI:** 10.3390/s24092927

**Published:** 2024-05-04

**Authors:** Isabella Luppi, Neel Pratik Bhatt, Ehsan Hashemi

**Affiliations:** Department of Mechanical Engineering, University of Alberta, Edmonton, AB T6G 1H9, Canada; luppi.isabella@gmail.com (I.L.); npbhatt@ualberta.ca (N.P.B.)

**Keywords:** distributed sensor networks, information filters, consensus filters, LiDAR-based state estimation, perception

## Abstract

A distributed state observer is designed for state estimation and tracking of mobile robots amidst dynamic environments and occlusions within distributed LiDAR sensor networks. The proposed novel framework enhances three-dimensional bounding box detection and tracking utilizing a consensus-based information filter and a region of interest for state estimation of mobile robots. The framework enables the identification of the input to the dynamic process using remote sensing, enhancing the state prediction accuracy for low-visibility and occlusion scenarios in dynamic scenes. Experimental evaluations in indoor settings confirm the effectiveness of the framework in terms of accuracy and computational efficiency. These results highlight the benefit of integrating stationary LiDAR sensors’ state estimates into a switching consensus information filter to enhance the reliability of tracking and to reduce estimation error in the sense of mean square and covariance.

## 1. Introduction

The evolution of distributed sensor networks (DSNs) and remote sensing has led to their application in motion planning and for the control of mobile autonomous systems for surveillance, environmental monitoring, warehouse management, and intelligent transportation [1,2,3,4,5,6].

Comprising spatially distributed sensors installed on infrastructures or mobile robots, DSNs can process local environmental data and generate a comprehensive scene overview through centralized or distributed state estimation [7,8,9,10,11], while maintaining asymptotic stability of the estimation error dynamics. As beneficial as these networks may be, the challenge is reliable estimation/sensing at the node (e.g., visual inference, point cloud clustering), particularly when it comes to target tracking in dynamic environments subject to partial or full occlusion [12,13,14,15]. Additionally, emerging research suggests that radar-based and UWB (Ultra-Wideband)-based distributed sensor networks offer promising alternatives for the localization and state estimation of mobile robots/vehicles [16,17], potentially enhancing accuracy and robustness in environments where LiDAR may encounter limitations.

The complexity and time-sensitivity of visual and LiDAR-based tracking of moving targets demand distributed observer design using continuous communication or event-triggered architecture [18,19,20,21,22], as accurate detection and pose estimation are integral for the robust and *scalable* tracking which is required for distributed motion planning and control of multiple mobile robots. In this regard, computationally efficient LiDAR-based state estimation at the edge (i.e., stationary sensing units), which provides accurate depth measurements provided by point clouds (removing the complexity of depth estimation and disparity map generation with a stereo/monocular camera), and maintains privacy (due to not processing of any images in the scene), has been the focus of the recent literature on remote estimation in DSNs [23,24,25,26].

However, processing the LiDAR point cloud data to obtain robots’ three-dimensional bounding boxes, including their headings, and removing outliers are challenging and have been addressed through geometrical (i.e., model-based), filter-based, and end-to-end learning methods [27,28,29]. Despite this, the inherent variability in mobile robots’/ground vehicles’ sizes and occlusion in dynamic scenes are the main challenges for reliability in detection and heading estimation in both model- and learning-based clustering and pose estimation approaches [30,31,32,33]. After reliable inference and pose estimation at the sensor node level, distributed estimation based on the Kalman consensus filter provides accurate estimates and convergence [34,35], depending on the topology of the networked sensors, guaranteeing the optimality of the variance and the stability of the error dynamics. Existing estimation methods in sensor networks, which centralize processing or use micro Kalman filters at each sensor node, impose considerable computational loads and require extensive inter-node communication during tracking and state estimation. These challenges are exacerbated in dynamic environments with multiple mobile robots and occlusion cases, impacting scalability and efficiency. Additionally, conventional L-shape fitting techniques for LiDAR-based tracking of mobile robots/vehicles are restricted to 2D data and necessitate sensors at the same vehicle height, limiting sensor placement flexibility and adaptability.

This paper develops a computationally efficient remote state estimation using a distributed architecture over LiDAR sensor networks, to address the limitations mentioned above and occlusion and limited visibility for the detection and tracking of mobile robots, through two key contributions:

- A generic LiDAR-based 3D bounding box detection and tracking method is designed to accommodate a wider range of sensor locations within DSNs;
- A distributed switching observer is designed to handle dynamic and occluded scenarios, to reduce overall computational cost, and to introduce short-term predictive capacity.

The remainder of the paper is organized as follows: Section 2 presents the background and point cloud processing. Section 3 introduces the designed distributed observer. Section 4 evaluates the performance and computational efficiency of the developed remote sensing framework in several experiments including occluded and dynamic scenes. Section 5 concludes this paper.

## 2. Background and Point Cloud Processing

**Figure 1:**
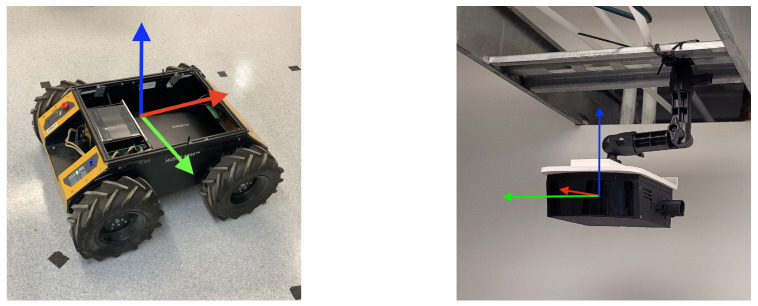
Experimental setup for evaluation of the LiDAR-based distributed state observer: unmanned ground vehicle (UGV) setup (**left**), and solid-state LiDAR used for remote sensing (**right**).

The position vector $p_{r}$ of a mobile robot centroid and the orientation $o_{r}$ of the bounding box of the tracked robot are defined by

(1) $$p_{r}={\left[\begin{array}{ccc} x_{r} & y_{r} & z_{r} \end{array}\right]}^{⊤},\;o_{r}={\left[\begin{array}{ccc} \phi_{r} & \psi_{r} & \theta_{r} \end{array}\right]}^{⊤},$$

where $x_{r}$, $y_{r}$, and $z_{r}$ represent the coordinates of the centroid in the fixed world frame {G}, while $\phi_{r}$, $\psi_{r}$, and $\theta_{r}$ are the bounding box (roll, pitch, and yaw) orientation angles. In order to develop the state observer and point cloud clustering, the following assumptions are made in this paper: **(i)** any variations in the z-value of the robot position are negligible (i.e., our case study is for the pose estimation and tracking of wheeled robots as shown in Figure 1); and **(ii)** the position $p_{s}$ and orientation $o_{s}$ of the sensor nodes are known in the global frame {G}. There is also no prior knowledge of the environment and dimensions of the robot. The computational challenge posed by high-dimensional point clouds is mitigated through voxelization (for dimensionality reduction). Given a point cloud $C\in R^{N\times 3}$, where *N* is the number of points, each represented by (x,y,z), the application of the Voxel Grid Filter can be represented as

(2) $$\begin{array}{c} C^{v}=\mathrm{VoxelGrid}\left(C,\alpha \right), \end{array}$$

in which α∈R is the parameter controlling the size of the voxels, and $C^{v}\in R^{N^{v}\times 3}$ is the resulting filtered point cloud with $N^{v}<N$ points. This divides the point cloud into a three-dimensional grid and selects a single representative point from each grid cell, discarding the rest. Each voxel $v_{i}$ is a cubic region of volume $\alpha^{3}$, and each point p∈C is assigned to a voxel based on its coordinates, determined by $\left(i_{x},i_{y},i_{z}\right)=\left(\left\lfloor \frac{x}{\alpha }\right\rfloor ,\left\lfloor \frac{y}{\alpha }\right\rfloor ,\left\lfloor \frac{z}{\alpha }\right\rfloor \right)$, where $\left(i_{x},i_{y},i_{z}\right)$ are the voxel indices in the grid corresponding to each point p, defining the voxel $v_{i}$ to which the point is assigned. All points p within each voxel $v_{i}$ are then represented by the centroid $c_{i}$, which is the arithmetic mean of the points in the voxel calculated by $c_{i}=\frac{1}{N(v_{i})}\sum_{p\in v_{i}}p$, where $N(v_{i})$ is the number of points in the voxel. This approach effectively reduces the data volume while preserving the essential structural features of the point cloud.

The point cloud data (obtained from the solid-state *Robosense* LiDAR shown in Figure 1) require transformation for alignment with the global frame {G}. The transformation of all points $p\in C^{v}$ involves rotation performed using a rotation matrix $R\in R^{3\times 3}$ derived from the Euler angles

(3) $$\begin{array}{c} R=R_{z}\left(\theta_{s}\right)\cdot R_{y}\left(\psi_{s}\right)\cdot R_{x}\left(\phi_{s}\right), \end{array}$$

where $R_{x}\left(\phi_{s}\right)$, $R_{y}\left(\psi_{s}\right)$, and $R_{z}\left(\theta_{s}\right)$ represent the rotation matrices around the *x*-, *y*-, and *z*-axes, respectively. The point cloud in the global frame is then expressed by

(4) $$\begin{array}{c} C^{G}=R\cdot C^{v}+t^{G}, \end{array}$$

in which $t={\left[\begin{array}{ccc} x_{s} & y_{s} & z_{s} \end{array}\right]}^{⊤}$ is the translation vector. Considering the first assumption, we employ a thresholding technique to identify and eliminate ground points from the point cloud as

(5) $$\begin{array}{c} C^{\prime }=\left\{p\in C^{G}\;|\;\;z>h_{f}+\epsilon \right\}, \end{array}$$

in which $C^{\prime }\in R^{N^{\prime }\times 3}$ includes the ground-less point cloud, $h_{f}$ is the elevation of the ground floor in the frame G, and ϵ is a slack variable to deal with potential noise or deviations from the ideal floor plane. While this approach effectively removes the majority of the ground points, it may also remove low-elevation non-ground points. Since the geometry of other operating robots in the environment is known and the aim of this research is accurate clustering and reliable distributed robot state estimation (not generating a cost/occupancy map), even the unexpected exclusion of some low-elevation points due to thresholding will not significantly affect the performance of the proposed framework.

### Clustering and Principle Component Analysis

To partition the point cloud into different groups, Euclidean clustering is employed. Given the point cloud $C^{\prime }$, Euclidean clustering involves finding the set of clusters $C_{c}=\left\{C_{1},C_{2},\ldots ,C_{k}\right\}$ that best represents the underlying structure of points $p\in C^{\prime }$, where each cluster $C_{i}$ is defined as a set of neighboring points that are close to each other in the 3D space, as in

(6) $$C_{i}=\left\{p_{j}|p_{j}\in C^{\prime }\;\mathrm{and}\;d_{i,j}\leq \rho \right\},$$

where i={1,…,k} denotes the cluster index, $j=\{1,\ldots ,N^{\prime }\}$ represents individual points within the point cloud, and ρ is a predefined distance threshold. It is important to note that the value of ρ must be at least as large as the leaf α of the voxels. If ρ≤α, no clusters will be formed due to the inability of points to meet the proximity criteria. Each cluster $C_{i}$ is characterized by computing the spans between the maximum and minimum values for height and length along the *x*- and *y*-axes. The selection of the clusters corresponding to the robot is achieved by comparing these dimensions to the prior geometry/size information of the robot operating in the environment. This comparison specifically considers the robot’s shortest side and its longest diagonal to ensure the dimensions fall within predefined thresholds.

By comparing the current clusters with the previous estimates, we associate each selected cluster (mentioned above) with the corresponding object through maximum likelihood data association. For each cluster $C_{i}\in C_{c}$, the association likelihood is calculated by evaluating the distance $d_{i}$ between the centroid of the cluster and the prior estimated position $p_{r}$. The cluster with the centroid closest to the past position estimate has the highest likelihood considering the bounds on the robot’s speed and acceleration. The chosen association is where $L_{i}$ is maximal, as in

(7) $$\begin{array}{c} i^{*}=\arg\max_{i}L_{i},\;L_{i}=\frac{1}{d_{i}}. \end{array}$$

The aim is to fit a 3D bounding box around each cluster of points obtained, accurately representing the shape and dimensions of the robot in the scene. The proposed method modifies the conventional L-shape fitting [36,37], which is effective when the robot’s point cloud consists of points belonging to the primary two edges of the moving robot. The LiDAR’s bird’s-eye-view estimated pose reveals the robot’s top in the point cloud.

**Figure 2:**
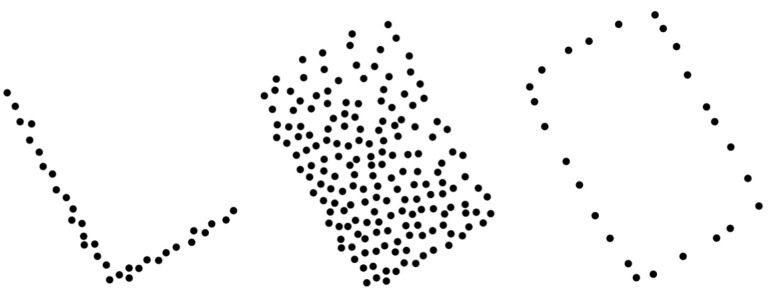
The xy-projection of the UGV robot’s clustered point cloud in real time for $z_{s}\leq z_{r}$ (**left**), for $z_{s}>z_{r}$ (**center**), and the convex hull of the cluster xy-projection (**right**).

To isolate the robot’s edges, the cluster $C_{i}$ is projected onto the xy-plane and encapsulated by a convex hull, $\mathrm{CH}(C_{i})$, which is the smallest convex polygon encompassing all points in cluster $C_{i}$ as shown in Figure 2. This method both identifies the mobile robot’s edges and regularizes its shape. If the robot shape is not a complete box (due to protruding wheels or a manipulator), the convex hull algorithm normalizes the estimated cluster edges into rectangles. Let $p_{h}$ denote each point on the convex hull, where h={1,2,…,N}, and *N* is the total number of points constituting the convex hull $\mathrm{CH}(C_{i})$. To classify the remaining points, we analyze the angle $\beta_{h}$ for each point $p_{h}\in \mathrm{CH}\left(C_{i}\right)$ with respect to the infrastructure-mounted LiDAR position $p_{s}$. By finding the lower bound $\beta_{\min}=\min\left\{\beta_{h}\right\}$ where $\beta_{h}=\tan^{-1}\left(\frac{y_{h}}{x_{h}}\right)$, the corresponding point $p_{\beta_{\min}}$ can be identified. Similarly, by finding the upper bound, $\beta_{\max}=\max\left\{\beta_{h}\right\}$, the point $p_{\beta_{\max}}$ can be determined. Hence, the points $p_{\beta_{\min}}$ and $p_{\beta_{\max}}$ correspond to the two primary edges of the mobile robot, as they represent the most laterally external points within the point cloud based on their angular positions.

**Figure 3:**
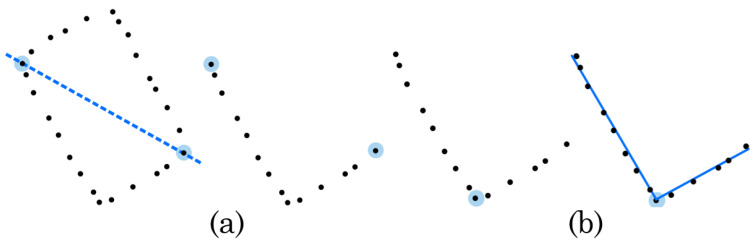
(**a**) Lateral-external points and L-shape reduction: (i) if $w_{j}\geq 0$, the point $p_{j}$ lies on the same side of the decision boundary (dashed blue line) as the sensor, or is collinear with the line segment defined by $p_{\beta_{\min}}$ and $p_{\beta_{\max}}$ (it is considered to be part of the main edges and is retained), and (ii) if $w_{j}<0$, the point $p_{j}$ is removed from $C_{i}$; (**b**) closest point and RANSAC line fitting.

To classify whether each $p_{h}\in \mathrm{CH}\left(C_{i}\right)$ belongs to the main edges, we examine the relative position of each $p_{h}$ with respect to the LiDAR position $p_{s}$ and the line segment defined by the two points $p_{\beta_{\min}}$ and $p_{\beta_{\max}}$. Initially, the vector v that links the two points is computed by $v=p_{\beta_{\max}}-p_{\beta_{\min}}$. Subsequently, the scalar product $w_{j}$ of the vector v and the vector from the sensor position $p_{s}$ to each point $p_{j}\in C_{i}$ is defined by $w_{j}=v\cdot \left(p_{j}-p_{s}\right)$. As schematically represented in Figure 3a, the scalar dot product $w_{j}$ is then used as an indicator of the position of $p_{j}$ with respect to $p_{s}$.

The nearest corner point $p_{n}$, which corresponds to the point with the minimum Euclidean distance between all points in the target robot and the sensor position $p_{s}$, is identified by

(8) $$\begin{array}{c} p_{n}=\arg\min_{p_{j}\in C_{i}}\;∥\;p_{j}-p_{s}∥. \end{array}$$

The cluster $C_{i}$ is then divided into two distinct subsets, namely $C_{i}^{A}$ and $C_{i}^{B}$, based on the angle each point forms with respect to the corner point $p_{n}$. The angle $\gamma_{j}$ between point $p_{j}$ and $p_{n}$ is computed by $\gamma_{j}=\tan^{-1}\left(\frac{y_{j}-y_{n}}{x_{j}-x_{n}}\right)$. Consequently, the points with $\gamma_{j}\leq \pi /2$ are included in the set $C_{i}^{A}$, while the remaining points are included in the set $C_{i}^{B}$, as in

(9) $$\begin{array}{c} C_{i}^{A}=\left\{p_{j}\in C_{i}|\gamma_{j}\leq \frac{\pi }{2}\right\},\;C_{i}^{B}=\left\{p_{j}\in C_{i}|\gamma_{j}>\frac{\pi }{2}\right\}. \end{array}$$

The lines $l_{i}^{A}$ and $l_{i}^{B}$ are then fitted to the point sets $C_{i}^{A}$ and $C_{i}^{B}$ using Random Sample Consensus (RANSAC) as shown in Figure 3b. The orientation difference between the two lines is denoted by

(10) $$\begin{array}{c} \delta \theta_{i}:=\left|\;\theta_{i}^{A}-\theta_{i}^{B}\right|, \end{array}$$

where $\theta_{i}^{A}$ and $\theta_{i}^{B}$ are the orientations of lines $l_{i}^{A}$ and $l_{i}^{B}$, respectively. If the difference δθ exceeds a predetermined threshold $\bar{\delta }\theta$, the lines $l_{i}^{A}$ and $l_{i}^{B}$ are accepted as the two edges of the target mobile robot. Consequently, the orientation of the robot, $\theta_{r}$, is determined by the longer of the two fitted lines, $l_{i}^{A}$ or $l_{i}^{B}$. The scale of the bounding box is then determined by

(11) $$\begin{array}{c} l_{r}=\max\left\{∥l_{i}^{A}∥,∥l_{i}^{B}∥\right\},\;w_{r}=\min\left\{∥l_{i}^{A}∥,∥l_{i}^{B}∥\right\}. \end{array}$$

If the angular difference δθ is within the threshold $\bar{\delta }\theta$, it suggests that points align along a single robot edge. This results in inaccurate bounding box identification using conventional L-shape detection methods. Afterwards, in the augmented LiDAR-based state clustering module (of the proposed estimation framework) in this paper, principal component analysis (PCA) is used to identify the bounding box’s major axis based on the first principal component of the point cloud transformed to a new coordinate system; the second and third components denote the bounding box minor axes. This transformation is achieved through the eigenvectors and eigenvalues of the cluster $C_{i}$ covariance matrix using $W_{i}\in R^{3\times 3}$ and

(12) $$\begin{array}{c} C_{\mathrm{PCA},i}=W_{i}C_{i},\;i=\left\{1,\ldots ,N^{\prime }\right\} \end{array}$$

in which $W_{i}=V\left(\frac{1}{N_{i}}C_{i}^{⊤}C_{i}\right)$ and V(·) denotes the eigenvector matrix of (·). The orientation $\theta_{r}$ of the robot in the new coordinates is given by the angle of the first principal component, as in $\theta_{r}=\tan^{-1}\left(\frac{W_{2,1}}{W_{1,1}}\right)$, where $W_{n,m}$ denotes the element in the *n*-th row and *m*-th column of the eigenvector matrix $W_{i}$. The length $l_{r}$ and width $w_{r}$ of the bounding box correspond to lengths of the bounding box along the *x*- and *y*-axes in the *new* coordinate system, respectively, and are determined as

(13) $$\begin{array}{c} l_{r}=\max\left(X_{\mathrm{PCA},1}\right)-\min\left(X_{\mathrm{PCA},1}\right),\\ w_{r}=\max\left(X_{\mathrm{PCA},2}\right)-\min\left(X_{\mathrm{PCA},2}\right) \end{array}$$

in which $X_{\mathrm{PCA},1}$ and $X_{\mathrm{PCA},2}$ denote the column number of $X_{\mathrm{PCA}}$.

## 3. Distributed Estimator Design

**Figure 4:**
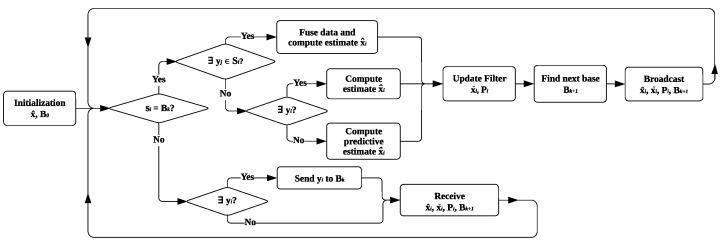
Overview of the distributed state observer on each node $s_{i}$ in the sensor network.

To achieve consistent robot pose estimates and reliable dynamic object tracking, a distributed estimation framework is designed in this section using a consensus filter. The designed consensus filter utilizes covariance dual-rate self-tuning, and provides a computationally efficient solution through the integration of short-term prediction and resource allocation in the distributed LiDAR sensor network for the estimation of robots’ states. The state estimation is conducted for each detected robot, allowing for the simultaneous tracking of robots’ states in dynamic environments. The architecture of the distributed state observer is provided in Figure 4 for each network node.

In this regard, the state variable vector of the dynamical system in discrete time is defined as

(14) $$\begin{array}{c} x\left(k\right)={\left[x_{r}\left(k\right),\;y_{r}\left(k\right),\;\theta_{r}\left(k\right),\;v_{x,r}\left(k\right),\;v_{y,r}\left(k\right),\;{\dot{\theta }}_{r}\left(k\right)\right]}^{⊤}, \end{array}$$

where $x_{r}\left(k\right),y_{r}\left(k\right)$, and $\theta_{r}\left(k\right)$ are the translational position of the robot’s centroid and its yaw angle (about the *z*-axis) in the world frame {G} at time k∈{0,1,2,…}, while ${\dot{x}}_{r}\left(k\right),{\dot{y}}_{r}\left(k\right)$, and ${\dot{\theta }}_{r}\left(k\right)$ are the translational velocities of the robot centroid and its yaw rate, respectively. The dynamic process in discrete time, which is used for the remote state estimation alongside the depth measurement in the distributed observer, is

(15) $$\begin{array}{c} x(k+1)=Ax(k)+Bu(k)+\vartheta (k), \end{array}$$

where ϑ(k) is the bounded noise of the process. This motion model is also used to address occluded scenes and intermittent cluster identification. The assumption of the constant acceleration is valid between two consecutive frames (within the sample time $T_{s}=50$ ms, which is sufficiently small for the robot maximum speed of $v_{x}=1.2$ m/s to warrant this approximation) and the acceleration is updated at every time step using a moving horizon forecasting method described at the end of this subsection. The state and input matrices then yield

(16) $$A=\left[\begin{array}{cc} I_{3} & T_{s}I_{3}\\ 0_{3} & I_{3} \end{array}\right],\;B=\left[\begin{array}{c} \frac{T_{s}^{2}}{2}I_{3}\\ T_{s}I_{3} \end{array}\right],$$

in which $T_{s}$ is the sample time between two consecutive frames, and I is the identity matrix. The sensor model of the stationary sensing unit *i* to track the state of the target/robot within the LiDAR sensor network with communication topology G=(V,E) is

(17) $$\begin{array}{c} y_{i}\left(k\right)=H_{i}\left(k\right)x\left(k\right)+w\left(k\right), \end{array}$$

where w(k) is the measurement noise of the sensor *i* and $H_{i}\left(k\right)$ is the observation matrix of the sensor *i*. Process and measurement noises within the distributed sensor setting are assumed to be uncorrelated, i.e., $E\left\{\vartheta_{k}w_{k}^{⊤}\right\}=0$. The input $u\left(k\right)={\left[a_{x}\left(k\right),\;a_{y}\left(k\right),\;{\ddot{\theta }}_{r}\left(k\right)\right]}^{⊤}$ has the perceived longitudinal/lateral translational and rotational accelerations of the clustered point cloud centroid, and is obtained over a moving horizon using position and speed gradients. In this regard, the acceleration input is estimated remotely using a moving horizon $n_{h}\in N$ over past estimated states, as in $u\left(k\right)≜E\left\{a_{k}\right\}$, where

(18) $$\begin{array}{c} a_{k}=\left\{\frac{d}{dt}{\dot{p}}_{r,q}\;:\;{\dot{p}}_{r,k}\in R^{3},\;k-n_{h}+1\leq q\leq k\right\}, \end{array}$$

where the finite difference of ${\dot{p}}_{r,k}={\left[v_{r,x},\;v_{r,y},\;{\dot{\psi }}_{k}\right]}^{⊤}$ over sample time $T_{s}$ is used to approximate the time derivative $\frac{d}{dt}{\dot{p}}_{r,k}$. For ease of notation, the updated values of the state variable and information matrices, which will be introduced in the next subsection, are denoted by $x^{+}$, and subscripts *k* are dropped. As a result, the process dynamics (15) and the sensor model of the stationary sensing unit *i* of the network are written as

(19) $$\begin{array}{c} x^{+}=Ax+Bu+\vartheta ,\;y_{i}=Hx+w_{i}. \end{array}$$

The motion model dynamically incorporates changes in the system response over time by continuously updating the control inputs for the consensus filter, and enables reliable tracking of the robot in the designed consensus filter in case of occlusion in dynamic environments.

### 3.1. Consensus Information Filter

The observer tracks the robot over the distributed sensor network, and the aim is for the network to provide a set of state estimates ${\hat{x}}_{i}\left(k\right)$ of the robot through the local exchange of messages among close/neighboring sensing units. At each time instance *k*, the state observer allocates the resource to only one stationary sensing unit denoted by the base node B(k) for state estimation in the global frame using a consensus information filter, in which the measurement $y_{i}$ (with covariance $R_{i}$) of the stationary sensing unit $s_{i}$ is used to calculate the information vector $z_{i}=H_{i}^{⊤}R_{i}^{-1}y_{i}$ and the information matrix $I_{i}=H_{i}^{⊤}R_{i}^{-1}H_{i}$. If the stationary sensing unit $s_{i}$, which has the robot in its field of view, matches the base node allocation B(k) for time *k*, it checks for any available measurements $y_{j}$ (with covariances $R_{j}$) from the following set:

(20) $$\begin{array}{c} S_{i}=\left\{j\;|j\in N_{i},\;l_{r,j}\geq {\bar{l}}_{r}\wedge w_{r,j}\geq {\bar{w}}_{r}\right\}, \end{array}$$

where $N_{i}$ denotes the neighbors of node *i* on the network (with topology G(k), which could be a dynamic/switching graph). If found, the sensing unit *i* receives the pair from the other sensing unit $j\in S_{i}$, and transforms them into the following information form:

(21) $$\begin{array}{c} {\bar{I}}_{i}=\sum_{j\in {\bar{S}}_{i}}I_{j},\;{\bar{y}}_{i}=\sum_{j\in {\bar{S}}_{i}}z_{j},\;{\bar{S}}_{i}=S_{i}\cup \left\{i\right\}. \end{array}$$

Then, the following distributed observer scalable in *n* (developed in [35,38]) is used to estimate the robot pose with ${\overset{ˇ}{x}}_{i}$ as the set of prior estimates (predictions) of the state x(k):

(22) $$\begin{array}{cc} {\hat{x}}_{i} & ={\overset{ˇ}{x}}_{i}+L_{i}\left({\bar{y}}_{i}-{\bar{I}}_{i}{\overset{ˇ}{x}}_{i}\right)+\xi P_{i}\sum_{j\in S_{i}}\left({\overset{ˇ}{x}}_{j}-{\overset{ˇ}{x}}_{i}\right),\\ {\overset{ˇ}{x}}_{i}^{+} & =A{\hat{x}}_{i}+Bu_{i},\;P_{i}^{+}=AL_{i}A^{⊤}+BQB^{⊤}, \end{array}$$

in which $L_{i}={\left(P_{i}^{-1}+{\bar{I}}_{i}\right)}^{-1}$ is the consensus information filter gain, ${\overset{ˇ}{x}}_{i}^{+}$ is the state update of the local information filter, and $\xi =\frac{\iota }{1+∥P_{i}∥}$. The small constant ι>0 is chosen in the order of the (integration) time interval $T_{s}$ used for discretization of the continuous-time constant-acceleration motion model. $∥P_{i}∥=\sqrt{\mathrm{tr}(P_{i}^{⊤}P_{i})}$ denotes the Frobenius norm of the matrix $P_{i}$. It should be mentioned that the distributed state observer in (22) is an information filter form of the Kalman consensus filter provided in the following:

(23) $$\begin{array}{cc} {\hat{x}}_{i} & ={\overset{ˇ}{x}}_{i}+K_{i}\left(y_{i}-H_{i}{\overset{ˇ}{x}}_{i}\right)+\eta_{i}\sum_{j\in S_{i}}\left({\overset{ˇ}{x}}_{j}-{\overset{ˇ}{x}}_{i}\right),\\ P_{i}^{+} & =AE_{i}A^{⊤}+BQB^{⊤}, \end{array}$$

with the update of the prior estimates ${\overset{ˇ}{x}}_{i}^{+}=A{\hat{x}}_{i}+Bu_{i}$, and the Kalman gain $K_{i}=P_{i}H_{i}^{⊤}{\left(H_{i}P_{i}H_{i}^{⊤}+R_{i}\right)}^{-1}$. The error covariance matrix (associated with ${\hat{x}}_{i}$) is denoted by $E_{i}=S_{i}P_{i}S_{i}^{⊤}+K_{i}R_{i}K_{i}^{⊤}$ with $S_{i}=I-K_{i}H_{i}$, where I is the identity matrix with proper dimensions.

**Remark 1.**  *The (global) asymptotic stability of the error dynamics of the Kalman consensus filter *(23)* with the choice of consensus gain $\eta_{i}=\xi S_{i}$ is proved in [35], Theorem 2, where all sensor node estimators asymptotically reach a consensus ${\hat{x}}_{1}={\hat{x}}_{2}=\cdots ={\hat{x}}_{n}=x$.*

In the case where no measurement is available through stationary sensors (i.e., $\bar{S}=\emptyset$), the state estimate is the predicted pose by the motion model (15), with u(k) as the behaviour-based input obtained over a horizon $n_{h}$ using (18). The base node B(k) for the next time step is selected based on a distance function $d(s_{j})$ that measures the Euclidean distance between the estimated state and the positions of the stationary sensing units:

(24) $$\begin{array}{c} d\left(s_{j}\right)={∥{\hat{x}}_{j}^{\left(1:2\right)}-p_{s_{j}}^{2D}∥}_{2},\;\forall j\in S \end{array}$$

where ${\hat{x}}_{j}^{\left(1:2\right)}≜{\left[{\hat{x}}_{r,j}\left(k\right),\;{\hat{y}}_{r,j}\left(k\right)\right]}^{⊤}$ includes the first two elements of the estimated state ${\hat{x}}_{j}$ in the 2D plane at time *k*. Then, the sensor node closest to the predicted state is chosen as the next allocated resource $B\left(k+1\right)≜s_{j}$, where $j=\arg\min\;d(s_{j})$. The stationary sensor $s_{i}$ broadcasts the updated and predicted state estimates, the updated covariance, and the base node B(k+1) for the next time step. The decision to allocate resources based primarily on spatial distance arises from the inherent spatial distribution observed among both the robot and the sensor nodes in the scene. This spatial distribution emerges as a result of factors such as the physical dimensions of robots and environmental constraints in dynamic scenes, which collectively ensure a dispersion of robots across the operational environment.

If sensor node $s_{i}$ is not assigned as the base node B(k), it functions as a contributing node within the network. Non-base nodes with available measurements transmit the measurement vectors $y_{j}$ and the corresponding measurement covariance matrices $R_{j}$ to B(k). Independently of their measurement status, all nodes subsequently receive the most recent state estimates $\hat{x}$, the prediction state $\overset{ˇ}{x}$, the covariance P, and the identifier $B_{k+1}$ of the next node scheduled for base status from $B_{k}$.

### 3.2. Covariance Dual-Rate Self-Tuning

The need for a dual-rate tuning method is that the noise in the proposed distributed state observer (due to LiDAR-based sensing and point cloud clustering) is time-varying and changes depending on the distance of the target vehicle/robot from the stationary sensing unit (i.e., the solid-state LiDAR sensor). Therefore, two initial covariances are used for the information filter: $\Sigma^{c}$, associated with $d^{c}$, which is the closest Euclidean distance (for close-range measurements); and $\Sigma^{f}$, associated with the farthest Euclidean distance $d^{f}$ from the sensing unit (for far-range measurements). The covariance allocation during the measurement update in the consensus filter is then a linear interpolation $R_{k}=a_{1}\Sigma^{f}+a_{2}\Sigma^{c}$, using the measured distance *d* (obtained by clustering and $l_{r},w_{r}$) with $\tilde{d}=d^{f}-d^{c}$.

## 4. Experimental Results and Discussion

**Figure 5:**
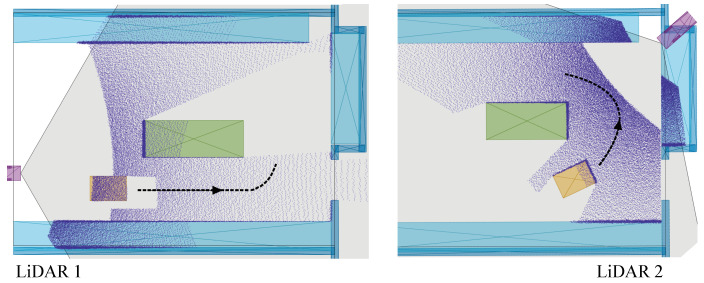
High-fidelity simulation of LiDAR coverage in indoor setting.

Several experiments were conducted in an indoor setting comprising various static and dynamic objects (with the operator walking around the robot) to evaluate the performance and computational efficiency of the developed LiDAR-based remote sensing and distributed estimation framework. The experimental methodology included testing in **(i)** full occlusion and **(ii)** partial occlusion in dynamic environments to evaluate the proposed distributed estimator’s accuracy, convergence, and computational efficiency using one and two LiDAR remote sensing units. Furthermore, high-fidelity simulations using Matlab (2023b) *Automated Driving* Toolbox were conducted for the **(i)** state estimation of multiple skid–steer mobile robot models using a network of solid-state LiDAR sensors (as shown in Figure 5), and **(ii)** identification of optimal coverage areas. The global frame of reference {G}, which located its origin at one of the corners of the testing hall/room, was established for all test scenarios.

The mobile test platform was a *Clearpath’s Husky* skid–steer UGV, with a maximum speed of 1.2 m/s for the conducted indoor tests in dynamic environments. The experiments employed a dual solid-state LiDAR setup (with horizontal and vertical fields of view of $120^{\circ }$ and $25^{\circ }$, respectively, a range of 150 m, a region of interest (ROI) feature for better resolution around fields of view limits, and accurate line separation, i.e., 3–4 cm detection range), each paired with an embedded *Jetson Xavier NX*. The first LiDAR was oriented at a pitch of $25^{\circ }$ and a yaw of $-90^{\circ }$; the second LiDAR had a pitch of $15^{\circ }$ and a yaw of $170^{\circ }$ degrees. This arrangement ensured comprehensive coverage of the area. The experimental methodology was designed to investigate two distinct scenarios (mentioned above), each tested five times in dynamic scenes under similar nominal control inputs to the UGV during navigation with obstacle avoidance. Throughout all testing scenarios, the UGV performed dynamic motion planning, as well as followed a consistent navigational path, and operated without any communication with the distributed sensor network.

The qualitative/quantitative experimental results are provided in the following subsections for two main scenarios: with only one stationary LiDAR sensor (to evaluate the clustering, tracking, and pose estimation), and with two remote sensing units. In this section, **(i)** “KCF-L” denotes the Kalman consensus filter architecture [35,39] which utilizes the proposed point cloud processing and pose estimation from Section 2; and **(ii)** “Obs.” denotes the developed consensus information filter, including point cloud processing and LiDAR-based distributed pose estimation (i.e., the whole pipeline proposed in this paper).

### 4.1. Occlusion with One Remote Sensing Unit

**Figure 6:**
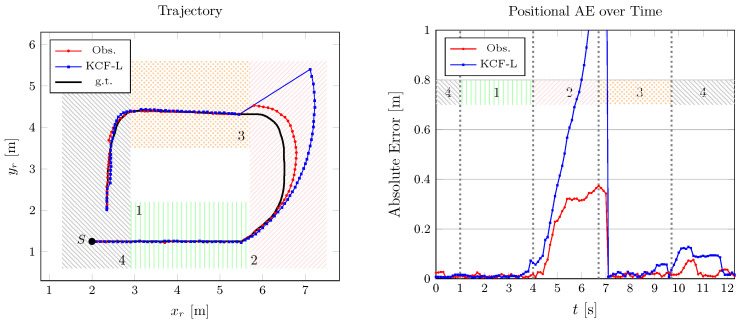
Tracked position and its absolute error over time in Scenario 1, where *S* is the starting point for the trajectory.

In the first test scenario, only LiDAR 1 was used for state estimation. The estimation results for this scenario are graphically illustrated in Figure 6. The results are consistent throughout the entire set of runs; therefore, only one run is displayed in the figure. Subsequently, the quantitative data presented are mean values computed from all runs.

**Figure 7:**
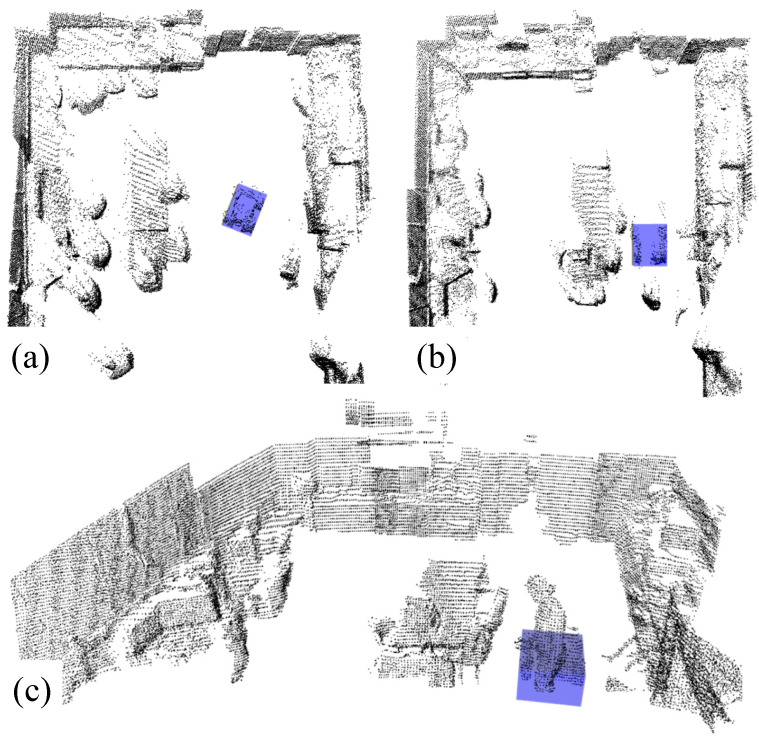
Point cloud clustering in (**a**) a dynamic scene with moving objects, (**b**) partial occlusion, and (**c**) full occlusion by an operator moving alongside the robot (in blue).

In the event of measurement unavailability, both the Kalman consensus filter (KCF-L) and the designed distributed observer use the predicted state as the estimation until the updated measurements for the subsequent step. However, KCF-L uses a general dynamics model without input estimation, resulting in constant velocities throughout the occluded area. Contrarily, the proposed framework estimates the input acceleration (for the dynamical process) using a moving horizon and the velocity magnitude right before the occlusion instance. This allows the designed framework to make accurate predictions of changes in the robot heading angle, thus reducing the estimation error after being updated by measurements from LiDAR 1. The point cloud representation of the scene partially occluded by a moving obstacle (i.e., operator) is shown in Figure 7, which confirms consistent clustering and state estimation using one remote sensing unit.

Position and orientation estimation errors are in Table 1, where MAE, MSE, and RMSE denote mean absolute error, mean squared error, and root mean square error, respectively. These values are mean quantitative results derived from the entire set of runs, which were five consecutive tests in the environment with dynamic objects and similar nominal control inputs (for the purpose of obstacle avoidance. The experimental studies confirm that as long as the robot trajectory does not change drastically between sample times for occluded scenes, the constant-acceleration motion model can be used for *full* occlusion.

Furthermore, it is important to note that the original bounding box L-shape fitting method fails to function under bird’s-eye-view conditions. This method was designed for sensors positioned at the same height as the vehicle; it effectively utilizes angled views to estimate dimensions and orientation.

### 4.2. Two Sensor Nodes

**Figure 8:**
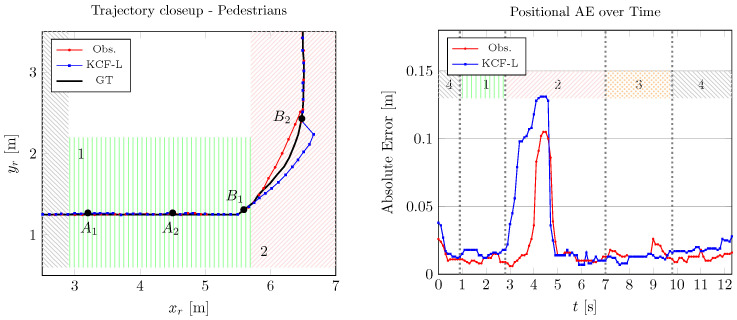
State estimation results and absolute error comparison with KCF-L for single run of Scenario 2, with full and partial occlusion.

In Scenario 2, the performance of the developed framework is evaluated using two sensor nodes. Initially, the environment does not present any significant occluding obstacles. Subsequently, the presence of the moving operator in Areas 1 and 2 leads to the formation of two occluded areas, discernible in Figure 8 between points $A_{1}$ and $A_{2}$ and between $B_{1}$ and $B_{2}$.

For occlusion A instances, the alteration is not significant as the robot maintains a straight path throughout the occlusion. In contrast, occlusion B presents more noticeable differences as the robot follows a curve and transitions into the second area, necessitating a shift in the sensors’ field of view (from $L_{1}$ to $L_{2}$). Although occlusion B incites an increase in the absolute error, the maximum estimation error by the proposed method does not exceed 11 cm for the position estimation, as shown in Figure 8.

The position and heading angle estimation results are compared in Table 2, which highlights improvements in the MSE, MAE, and error covariance using the proposed framework. Even though the performances of both state observers are very close for this scenario, under full robot visibility, Obs. demonstrates superior performance under pedestrian occlusion. This is attributed to the input estimation, which is a feature not employed by the KCF-L.

Several experimental tests and error convergence analyses in various high-/low-excitation maneuvers with occlusion provide insightful observation on the impact of perceptually degraded conditions on the performance of the designed LiDAR-based distributed switching state observer in this paper: **(i)** When the robot is fully visible by SSUs, the developed observer and the KCF-L (which uses the developed computationally efficient clustering approach, but utilizes the conventional Kalman consensus filter) perform reliably for position estimation; however, less heading estimation error for “Obs.” offers an advantage in terms of computational cost on a network level, making it a more efficient choice in scenarios where computational resources are a limiting factor. **(ii)** The variance in the estimation error by the proposed framework is lower than that of KCF-L in almost all experiments. This is attributed to the utilization of the region of interest for outlier identification/rejection in the new distributed estimation framework.

The solid-state LiDAR used for experiments features accurate line separation at a 3–4 cm detection range, generating accurate point clouds with high frequencies of 50 Hz in indoor settings. The proposed distributed estimation framework enables processing and publishing the pose estimates with a sample time $T_{s}=50$ ms. Considering the maximum robot speed of $v_{x}=1.2$ m/s, the maximum range of 70 m for indoor operation, and the sampling time of 50 ms (including the process required for clustering/estimation), the utilized LiDAR enables accurate estimation (RMSE < 13 cm even with occlusion) with various robot speeds. For other outdoor settings with higher vehicle speeds, e.g., intelligent transportation, the correlation between the speed and accuracy of LiDAR point clouds is an important aspect and needs to be taken into account.

**Figure 9:**
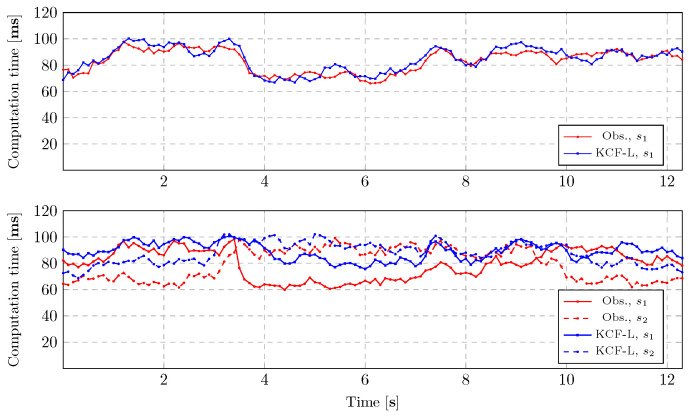
Computational time of the proposed LiDAR-based remote sensing framework for Scenario 1 (**top**) and Scenario 2 (**bottom**) subject to occlusion and dynamic objects moving around the robot.

The computational times of the proposed distributed state observer are shown in Figure 9 for both scenarios (with occlusions) using a 6-core NVIDIA Carmel ARM 1.9 GHz with 16 GB memory (available through *Jetson Xavier NX* embedded systems for edge computing purposes), and confirm the computational efficiency of the framework, i.e., <100 ms, which is sufficient for the accurate motion planning of mobile robots with maximum speeds of 2 m/s, and is important for scalability in larger sensor networks.

## 5. Conclusions

In this paper, a remote LiDAR-based state estimation (and tracking) framework is developed and experimentally validated for mobile robots in distributed sensor networks by designing a consensus information filter and computationally efficient point cloud processing. As confirmed by experiments, the proposed approach hinges on two primary contributions: a LiDAR-based 3D bounding box detection and tracking method that expands the versatility of sensor placements in distributed sensor networks, and a node-switching distributed observer to address occlusion and uncertainties in dynamic environments, achieving average estimation errors as low as 2 cm and $2^{\circ }$ under nominal conditions (<4 cm and <$3.5^{\circ }$ under occlusions). These advancements significantly reduce computational demands (i.e., less than 100 ms) while incorporating short-term motion predictive capabilities, thereby enhancing the scalability and efficiency of mobile robot tracking in dynamic scenes with human presence while maintaining privacy. Through experimental evaluations and high-fidelity simulations in indoor settings, the effectiveness of the framework in terms of the accuracy of the estimation and the asymptotic stability of the error dynamics is demonstrated in normal and arduous scenarios (including occlusion, limited visibility, and dynamic obstacles) and compared with the Kalman consensus filter. Future work will focus on further refining the predictive modeling in large sensor networks and exploring its applications in unstructured operational settings with multi-robot tracking. Additionally, research will aim to move beyond the assumption of flat terrain and negligible z-axis variations in robot positions to handle more complex and varied terrain for field applications with stationary sensing units. 

## Figures and Tables

**Table 1:** Position/heading estimation errors in Scenario 1.

**Method**	**MAE (m)**	**MSE**	**RMSE**
Obs.	0.074	0.019	0.138
KCF-L	0.199	0.192	0.438
**Method**	**MAE** **(°)**	**MSE**	**RMSE**
Obs.	5.2	64.3	8.0
KCF-L	12.5	509.4	22.6

**Table 2:** Position/heading estimation errors in Scenario 2 with 2 remote sensing units and occlusion scenarios.

**Method**	**Pedestrians**	**MAE (m)**	**MSE**	**RMSE**
Obs.	No	0.018	0.001	0.032
KCF-L		0.018	0.001	0.031
Obs.	Yes	0.023	0.002	0.042
KCF-L		0.031	0.003	0.052
**Method**	**Pedestrians**	**MAE** **(°)**	**MSE**	**RMSE**
Obs.	No	1.9	5.9	2.4
KCF-L		2.0	5.8	2.4
Obs.	Yes	3.4	10.2	3.2
KCF-L		4.9	33.8	5.8

## Data Availability

Data are contained within the article.

## Abbreviations

The following abbreviations are used in this manuscript:

LiDAR	Light detection and ranging.
DSN	distributed sensor networks.
PCA	principal component analysis.
